# FASTQSim: platform-independent data characterization and in silico read generation for NGS datasets

**DOI:** 10.1186/1756-0500-7-533

**Published:** 2014-08-15

**Authors:** Anna Shcherbina

**Affiliations:** Department of Bioengineering Systems and Technologies, MIT Lincoln Laboratory, 244 Wood St, 02421 Lexington, MA USA

**Keywords:** Simulator, Algorithm, Next generation sequencing, FASTQ

## Abstract

**Background:**

High-throughput next generation sequencing technologies have enabled rapid characterization of clinical and environmental samples. Consequently, the largest bottleneck to actionable data has become sample processing and bioinformatics analysis, creating a need for accurate and rapid algorithms to process genetic data. Perfectly characterized *in silico* datasets are a useful tool for evaluating the performance of such algorithms. Background contaminating organisms are observed in sequenced mixtures of organisms. *In silico* samples provide exact truth. To create the best value for evaluating algorithms, *in silico* data should mimic actual sequencer data as closely as possible.

**Results:**

FASTQSim is a tool that provides the dual functionality of NGS dataset characterization and metagenomic data generation. FASTQSim is sequencing platform-independent, and computes distributions of read length, quality scores, indel rates, single point mutation rates, indel size, and similar statistics for any sequencing platform. To create training or testing datasets, FASTQSim has the ability to convert target sequences into *in silico* reads with specific error profiles obtained in the characterization step.

**Conclusions:**

FASTQSim enables users to assess the quality of NGS datasets. The tool provides information about read length, read quality, repetitive and non-repetitive indel profiles, and single base pair substitutions. FASTQSim allows the user to simulate individual read datasets that can be used as standardized test scenarios for planning sequencing projects or for benchmarking metagenomic software. In this regard, in silico datasets generated with the FASTQsim tool hold several advantages over natural datasets: they are sequencing platform independent, extremely well characterized, and less expensive to generate. Such datasets are valuable in a number of applications, including the training of assemblers for multiple platforms, benchmarking bioinformatics algorithm performance, and creating challenge datasets for detecting genetic engineering toolmarks, etc.

**Electronic supplementary material:**

The online version of this article (doi:10.1186/1756-0500-7-533) contains supplementary material, which is available to authorized users.

## Background

The advent of high-throughput sequencing technologies has enabled rapid characterization of clinical and environmental samples. The next generation sequencing revolution has seen the rise of multiple instruments and methodologies. With the decreasing cost of sequencing technology, sample processing and bioinformatics analysis pose the largest bottleneck to actionable data for critical medical and defense applications [1]. A need exists for more accurate and rapid algorithms to process genetic data. Quantitative evaluation tools are needed to measure the performance of these algorithms. One approach to measuring algorithm accuracy involves executing the algorithm on *in silico*-generated datasets, which consist of a host backbone “spiked” with reads from other organisms and plasmids in known concentrations. Since the exact quantity of reads and genes from each source in the composite dataset is known, the degree to which an algorithm can report the dataset composition serves as a measure of the algorithm’s performance [2–4].

Numerous tools exist to characterize NGS datasets and use the resulting profiles to generate *in silico* data, but many of these are platform-specific (Table 1). This limits their applicability, since NGS platforms differ in read lengths and error profiles [5]. For example, the Roche 454 platform produces 400 base pair reads with a 99% accuracy level,while Pacific Biosciences has developed a platform that produces 10000 base pair reads at a 90% accuracy level. Additionally, sequencing platforms exhibit differing rates of insertions and deletions. The rate of short tandem repeat indels, as opposed to random indels, also differs by platform. Finally, for some platforms, such as PacBio, errors are randomly distributed throughout a read, whereas others, like IonTorrent, tend to accumulate more errors toward the end of a read [6, 7]. Even among datasets generated with the same platform, the choice of polymerase can affect the probability of a single mutation from a given base to a different base.

**Table 1:** **Popular** ***in silico*** **read simulators**

Technology	Supported platforms	Capabilities	Limitations
PBSim [9]	PacBio	Simulates both continuous long reads (CLRs) and circular	Limited insertion and deletion (indel)
		consensus sequences (CCS); supports sampling-based	support
		simulation (in which both length and quality scores are	
		sampled from a real read set) and model-based simulation	
FlowSim [8]	Roche 454	Simulates read length and quality in flow space	No indel support
dwgsim [10]	Illumina, IonTorrent	Whole-genome simulator	Uniform read length
ART [11]	Roche 454, Illumina Solexa	Read error model, quality profiles	No simulation of indels for short
			tandem repeats (STRs)
Maq [12]	Illumina Solexa	Single nucleotide polymorphism (SNP) simulation	Fixed mutation rate does not model
			real-world data
Grinder [13]	Platform-independent	Shotgun or amplicon read libraries	Limited indel support
MetaSim [14]	454, Illumina, Sanger	Simulation, assembly, mapping	Does not assign quality values to reads
GemSim [15]	454, Illumina	Simulation	Fixed length and mutation rates

Among *in silico* data generation tools that provide the capability to simulate read length and quality scores according to a user-specified distribution, many do not provide extensive support for generating insertions, deletions, and single base substitutions. The datasets generated by these tools consequently do not match instrument error profiles. For example, the GemSim tool uses a fixed length, fixed mutation rate model to simulate reads for the Roche 454 and Illumina sequencing platforms. Other tools, such as Flowsim *in silico*, generate data that is consistently better in terms of contig size than assemblies of real data. This is due to the fact that Flowsim generates all simulated reads from the reference genome in a manner such that 100% of the reads map back to the genome, whereas for real datasets generated with Roche 454, the percentage is closer to 98.7% [8]. Other tools, such as ART and PBsim, do match the insertion/deletion (indel) profiles of NGS sequencers, but do not simulate short tandem repeat (STR) insertions or deletions. For example, in the case of PBsim, the deletion probability is uniform throughout all positions of every simulated read, which is computed from the mean error probability of the read set and the ratio of differences [9]. This doesn’t model actual sequences, where indel rates vary in accordance with base position and other factors, such as repeat size. In summary, no current state-of-the art tool is able to generate *in silico* data that is completely indistinguishable from datasets generated by the intended target sequencing platform.

The FASTQSim software package was developed to characterize NGS datasets in FASTQ format and generate *in silico* reads with matching error profiles from reference sequences. FASTQSim is platform-independent and provides functionality to create spiked datasets by adding one or more organisms and plasmids to an existing dataset. It supports introduction of known sequence variants and allows for the creation of synthetic datasets that can be used for evaluation of bioinformatics tools.

## Methods

The FASTQSim software package consists of two main components: *FASTQcharacterize* and *FASTQspike*. The tool FASTQcharacterize is used to analyze error profiles of existing datasets. The outputs of the FASTQcharacterize tool serve as inputs for the FASTQspike tool, which uses the supplied error profile to generate *in silico* reads and blend target sequences into a background dataset. Error profiles for common NGS platforms, including IonTorrent, Roche, PacBio, and Illumina, are included included with the software and can be used directly with FASTQspike without the need to run FASTQcharacterize first.

### Dataset error profile characterization with the FASTQcharacterize tool

**Figure 1:**
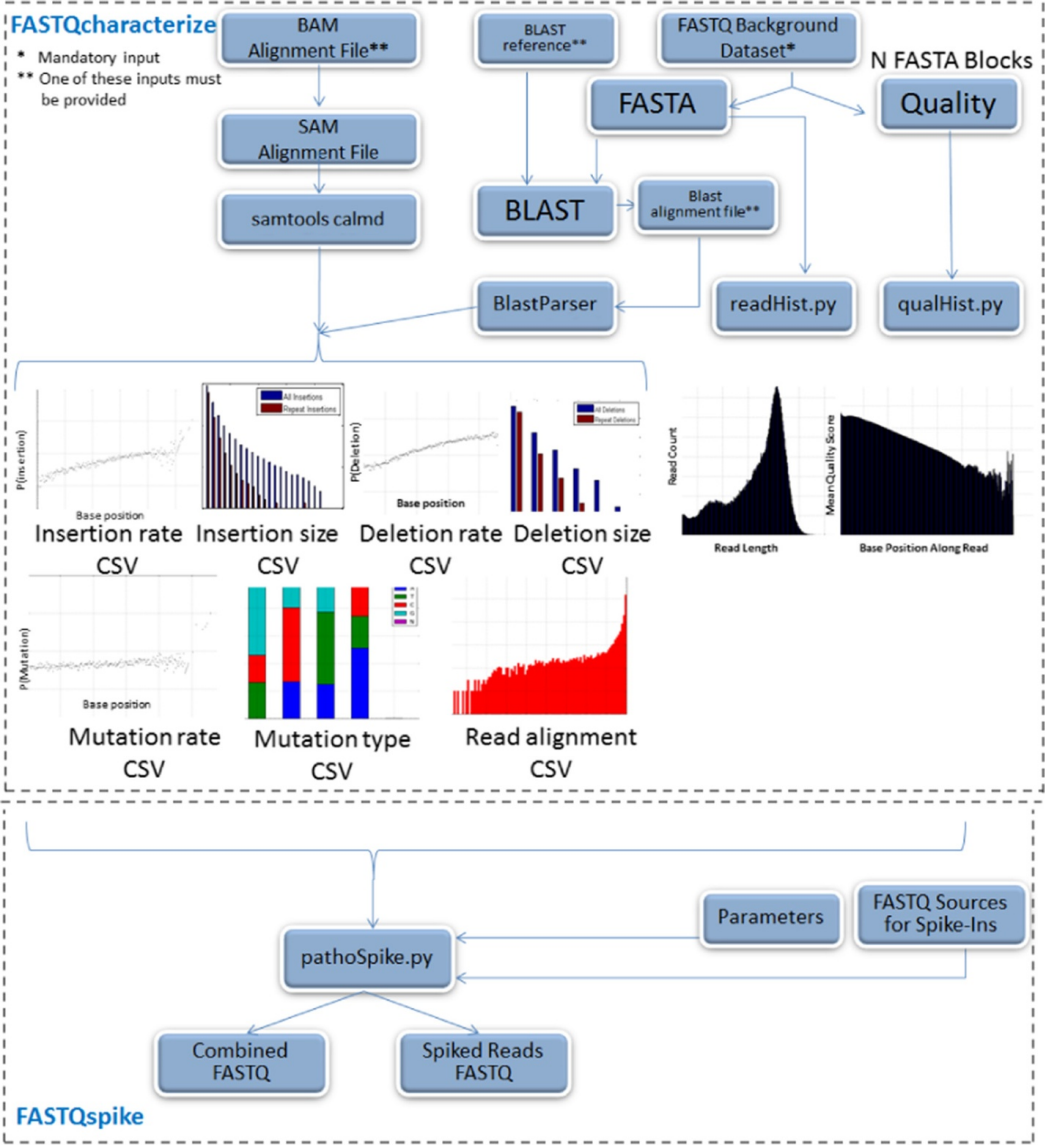
**FASTQSim pipeline.**

The data characterization pipeline is illustrated in Figure 1. Inputs to the characterization pipeline include: 1. a target file to be characterize (FASTQ format)

2. one of the following:
  - reference file (FASTQ format)
  - a BLASTn output file for the target dataset against the reference of interest
  - a SAM/BAM alignment file of the target against the reference

In the included example, a dataset from sequenced human blood was used with the human genome reference. A read length distribution was generated empirically from the input FASTQ file. A distribution was also generated for quality scores as a function of base position along a read. The FASTA file was split into blocks for parallel processing (default block size is 1000 reads, but this parameter is customizable). BLASTn is used to align these blocks with the reference genome [16]. By default, five blocks from the FASTA file were sampled at random, since it was experimentally observed that 5000 reads is generally sufficient to characterize most datasets. However, the FASTQcharacterize tool can be customized to sample any number of blocks with an option in the parameter file. An externally generated BLASTn result file can also be supplied to the tool for characterization. The Java BlastParser tool was then used to compare query reads to matching target sequences in the reference genome [17]. This tool is available as a compiled Java jar file in Additional file 1.

As an alternative to using BLAST for alignment, a BAM/SAM file can be used as an input to the algorithm, skipping the initial alignment step. Consequently, any NGS aligner that generates a BAM/SAM file can be utilized with FASTQcharacterize as an alternative to BLAST. A multiple sequence alignment was performed for each set of overlapping reads. Insertion, deletion, and mutations probabilities were computed for each aligned base in the target sequence. A probability distribution for repeat insertions and deletions was generated, taking into account repeat size. Finally, instrument error was quantified by computing a distribution for the fraction of bases in source file reads that aligned to reads from the target database. These values were aggregated across all aligned reads to determine a sample profile.

**Figure 2:**
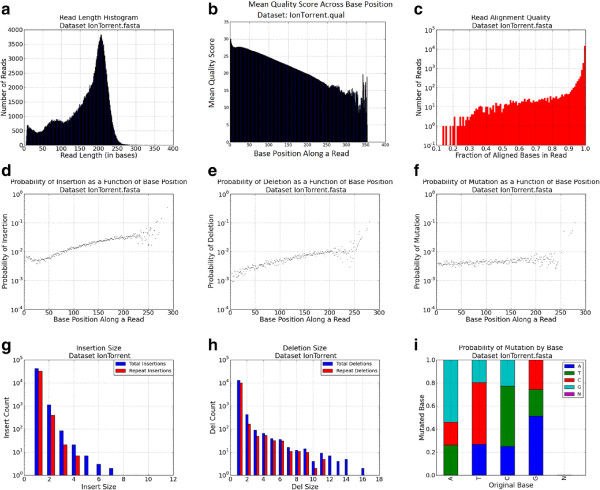
**IonTorrent dataset characterization profile.**

An example profile for an IonTorrent dataset is presented in Figure 2. The FASTQcharacterize tool generates a set of nine comma-separated-value files and accompanying images that constitute the dataset profile. The output files characterize: 1. Read length

2. Quality scores
3. Insertion rates
4. Deletion rates
5. Insertion size distribution
6. Deletion size distribution
7. Single point mutation rates
8. STR indel frequencies for repeats with *2, 3, …, n* repeated bases
9. Quality of read alignment to the reference

### Blended data generation for in silico projects with the FASTQspike tool

The FASTQspike tool uses the outputs of FASTQcharacterize to generate an *in silico* dataset. The tool provides the option to generate an *in silico* dataset from scratch or to add *in silico* reads to an existing background dataset. The reads are aggregated into an output FASTQ file in a manner such that the *in silico* reads are indistinguishable from the experimentally-generated background data. An additional FASTQ file that consists solely of spiked reads is also generated.

**Figure 3:**
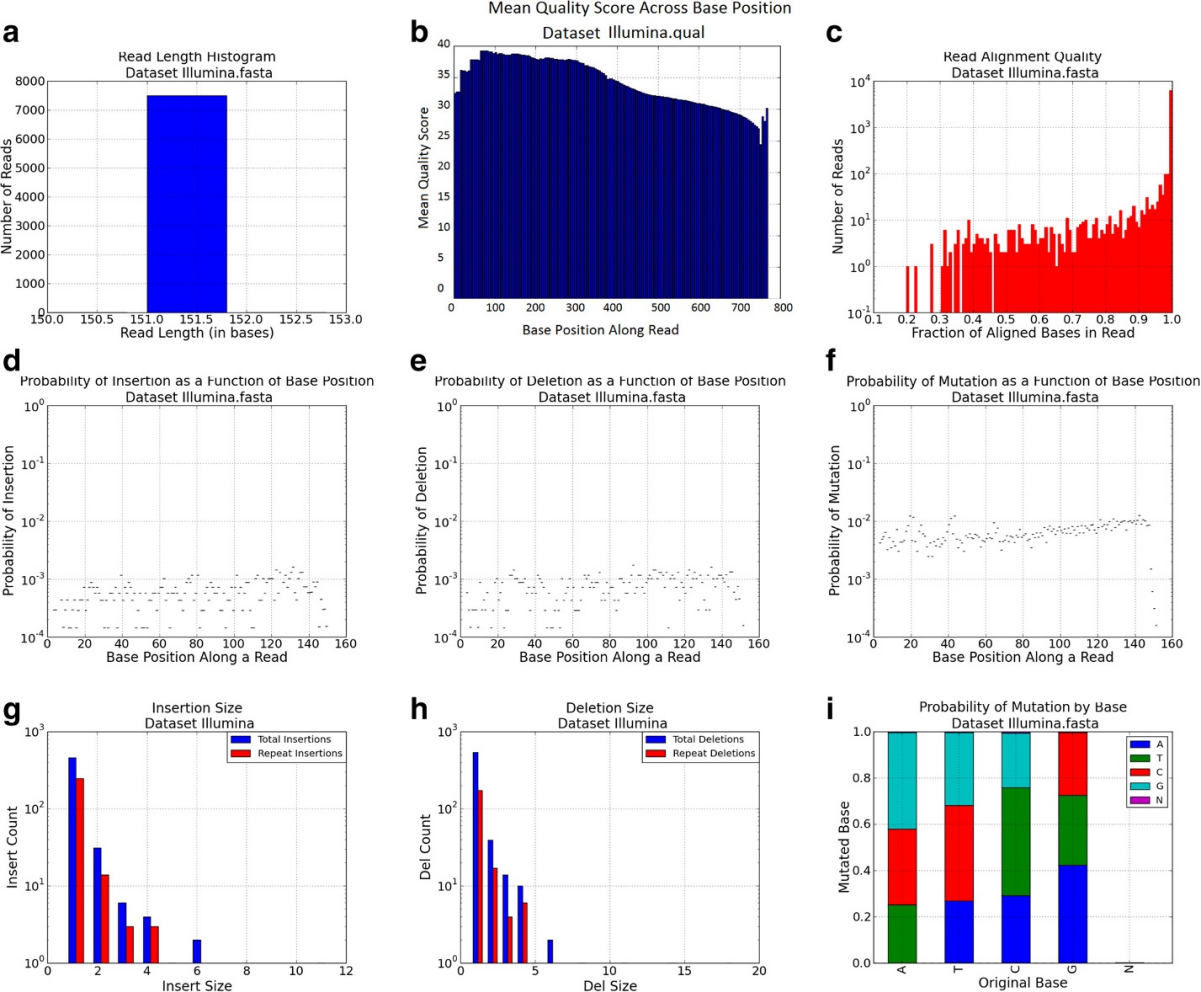
**Illumina dataset characterization profile for human whole blood sample.**

**Figure 4:**
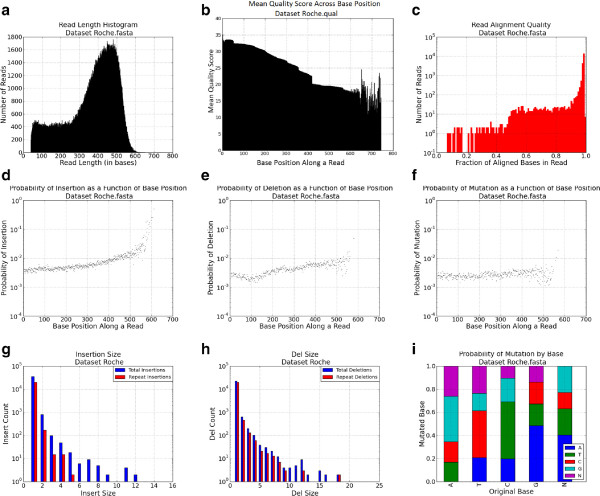
**Roche dataset characterization profile for human whole blood sample.**

Inputs to the FASTQspike pipeline include:

1. Background FASTQ. This input is optional. It is also possible to run FASTQspike without supplying a background file.
2. Background characterization profile. Profiles for common NGS platforms are included iwth teh FASTQsim distribution. These are described in Figure 2 (IonTorrent), Figure 3 (Illumina), Figure 4 (Roche), and Figure 5 (PacBio). Customized profiles can be generated with the FASTQcharacterize tool. The ability to generate customized read length, quality, and error distribution profiles enables a platform-independent approach to *in silico* data simulation.
  - Read length distribution

**Figure 5:**
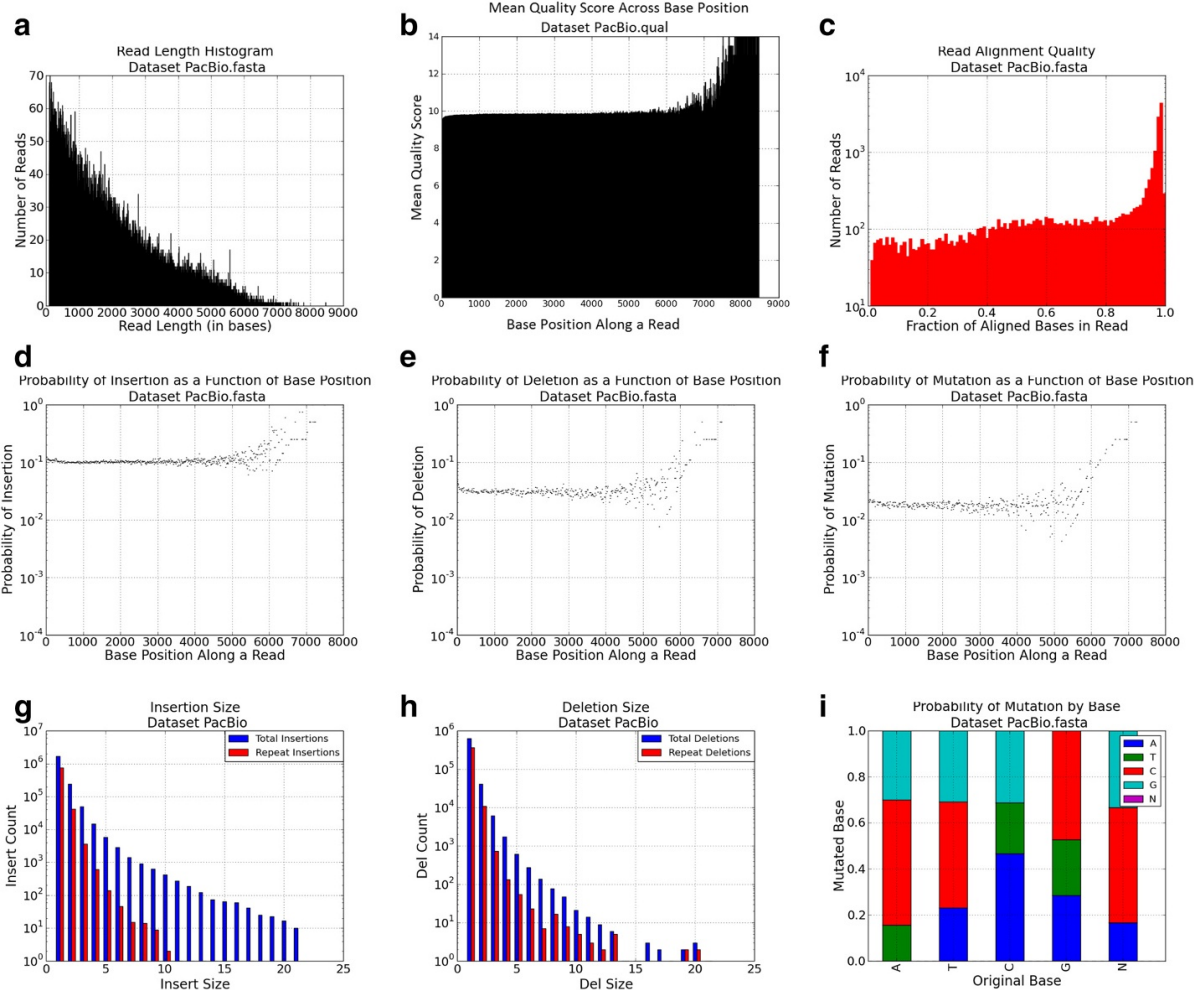
**PacBio dataset characterization profile for human whole blood sample.**
  (b) Quality score distribution
  (c) Instrument-specific insertion and deletion rates
  (d) Insertion and deletion size distribution (repeats)
  (e) Instrument-specific mutation rates
3. For each target organism
  - FASTA file with source sequence material. The indel and single point mutation rate profiles listed above refer to instrument-specific values.
  - Target genome/plasmid morphology (circular/linear)
  - Desired mean coverage

The first phase of the FASTQspike algorithm consists of generating reads for the target organisms so that the specified coverage is achieved. While target coverage is less than the specified value, the algorithm randomly selects a read start position and strand orientation in target genome to ensure realistic non-uniform target coverage. It also randomly selects read length from underlying background distribution. A parameter is used to specify minimum sequence length. This value is set to 50 by default, but can be altered by the user. Based on user-specified parameters, the algorithm can treat the source FASTQ as either a circular or linear DNA molecule, and reads are generated accordingly. For a circular plasmid genome, a read that runs off the end of the source DNA molecule will wrap around to the beginning, whereas a read from a linear genome will be truncated if it is too long. Each template spiked sequence is generated to have twice as many bases as the final desired sequence length. This provides a buffer region for the error profile matching step.

Once a set of sequences has been simulated from the target FASTA input, these sequences are modified to simulate sequencing errors. The probability distributions of these errors are generated to mimic the background distribution for deletions, insertions, and single point mutations. Insertions and deletions may consist of one or multiple base pairs. Indels may further be subdivided into two kinds: a random base indel or a short tandem repeat (STR) indel. Both random and STR indels are generated at a rate that matches the supplied error profile for the background dataset. As part of this process, linear regression is used to account for noise effects. Linear regression is also used to interpolate for missing or incorrect data points in the data characterization profiles.

**Figure 6:**
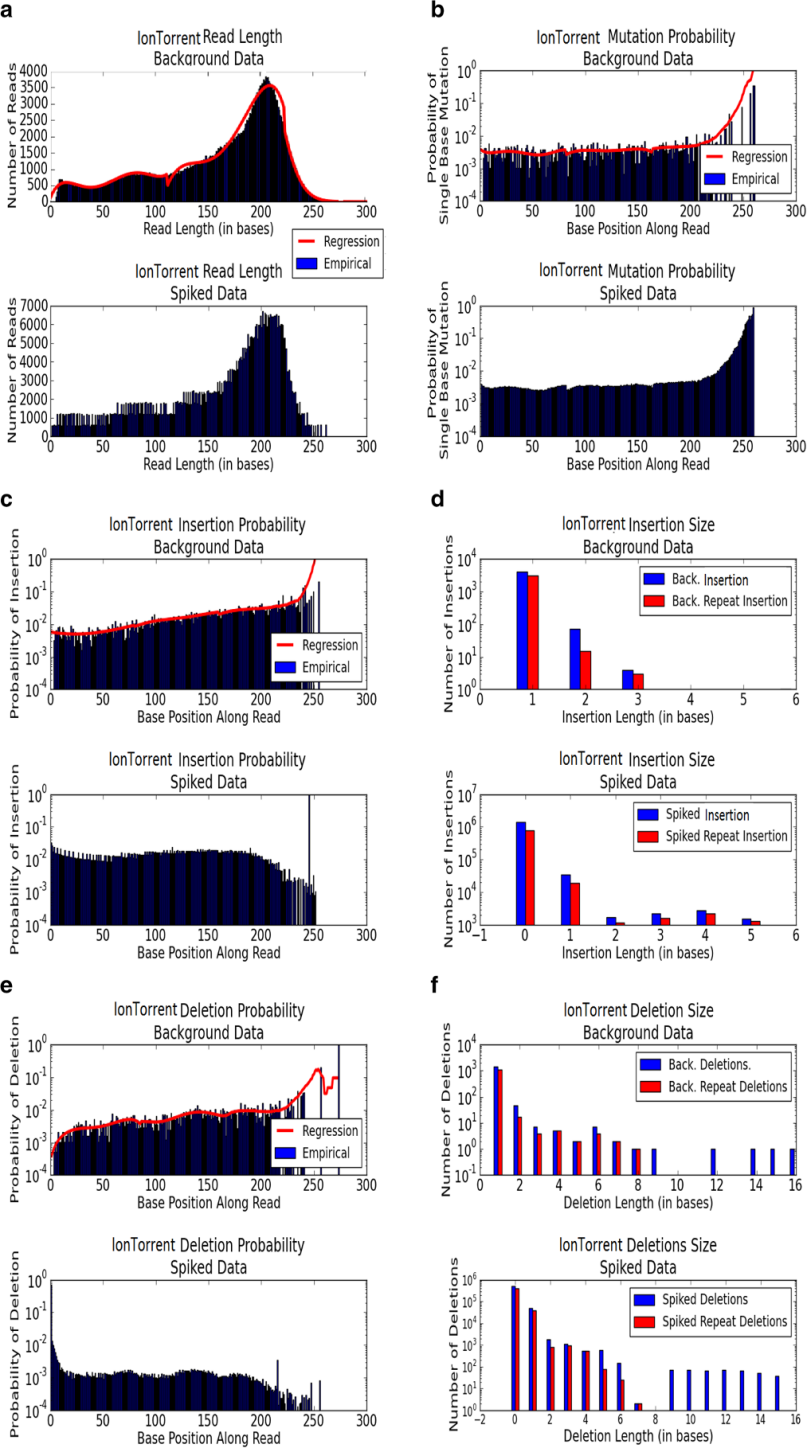
**FASTQspike curve fitting for IonTorrent human whole blood sample dataset.** Several types of functions are fit to the empirical data, and least squares linear regression is used to determine the curve of best fit.

Since dataset profiles are generated by aggregating statistics across a number of reads, characterizations are less accurate where there are fewer datapoints. This is particularly relevant for calculated indel and single base mutation rates for base pair positions toward the end of the sequence. Figure 2a indicates that relatively few reads of length greater than 250 bases are present in the dataset. As a result, in Figures 2d, 2e, and 2f the statistics for base pair positions 250 and higher are derived from a small sample size. Consequently, these values were not used for spiking *in silico* reads, but a curve-fitting strategy was used instead to model indel and single point mutation rates for high base pair positions. If a spiked read has a length within the lower 95% of the observed range (within two standard deviations above the mean), the empirical distribution data is used to simulate insertions, deletions, and mutations. Least squares regression is used to fit a function to the empirical data distribution for longer reads. The best curve fit is selected from multiple functions, including the linear, 2-degree polynomial, 3-degree polynomial, 4-degree polynomial, 5-degree polynomial, power, exponent, sum of exponents, logarithm, or Gaussian function. Each type of curve is fit to the empirical data, and the curve of best fit is determined via least squares regression. This curve is then used to interpolate missing datapoints within the empirical range, which may result if few reads of a specific length are present. For example, in Figure 6, there is a sharp drop in indel rates for base 950 and up, due to low numbers of reads in the dataset of that length. The linear regression model is also used to extrapolate to obtain values for datapoints in the high base pair range (Figure 6). This adaptive model is unique to FASTQsim and allows for simulation of platform-independent dataset specific read length distributions and error rates.

The mean and standard deviation of the number of deletions per read for the background data is calculated. These parameters are used to fit a Gaussian distribution for the number of deletions in a read. This distribution is sampled to determine the number of deletions to perform for each read. If this number is non-zero, the deletion sizes are determined by randomly sampling from the deletion size distribution generated in the prior step. Next, statistics about the probability of deletion for a repeat of a given size are used to decide whether the deleted bases will contain a repeated sequence. If the sequence does not contain a repeat of the appropriate length, the repeat length is shortened by one base and the sequence is re-examined. This is repeated until a repeat of the determined size is located within the sequence, or the repeat count is reduced to 1. If a non-repeat is to be deleted from the sequence, the position of the deletion is determined by sampling from the deletion probability distribution.

Insertions and single base pair mutations are handled in the same manner as deletions. If a repeat is to be inserted, a position is determined from the insertion probability distribution, and a series of bases at that position are selected as the repeat unit to insert. If a non-repeat is to be inserted, the bases to insert are generated at random, with equal probability for each of the four bases. At the end of this phase, the spiked sequence is clipped to the desired length.

Finally, if a background FASTQ file is provided, the spiked reads are inserted into the background FASTQ file at random locations. Slices of the background FASTQ file are read into memory, each of which is 100 MB in size. A subset of the generated reads are chosen at random to spike into that portion of the file. The quality scores for each read in the background are separated into groups by the length of the read. For each spiked read, a quality string matching the length of this read is selected from the appropriate group. Read names are assigned to the spiked reads by randomly selecting a position in the background FASTQ file to insert the read and “interpolating” the read names of the prior and following sequence.

The result is a blended FASTQ dataset that consists of both the background data and simulated reads, and is indistinguishable from the background data. As illustrated in Figure 6, the characterization profile of the blended dataset is identical to that of the source dataset.

## Results and discussion

### Speed and memory

To test FASTQSim’s speed and memory, the example whole blood IonTorrent dataset was characterized with the FASTQcharacterize tool. The dataset contains 319,365 reads, with a mean length of 160 bases. The characterization software ran in 13 min., 40 s. and utilized an average of 1.2 GB of RAM. The dataset was then spiked with E. coli str. K-12 substr. MG16555 [Genbank:NC_000913.2] at a mean coverage of 20 ×. BioBrick cloning vector pSB3C5-I52001 [Genank:EU496103.1] reads were spiked into the same dataset at a coverage of 200 ×. The NGSpike software is parallelized for multi-threaded execution. Executing the software with a single thread resulted in a runtime of 3 h., 24 min., 10 s. and utilized an average of 1.6 GB of RAM. Increasing the number of threads to 30 reduced led to a runtime of 1 hour, and further increasing the number of threads to 100 allowed for code execution in 30 minutes. A logarithmic drop in runtime was thus observed by assigning additional threads for NGSpike execution. Algorithm runtime scales linearly with datasets size. The software was run on a Linux machine with a 1400 MHz CPU running CentOS 5.

### Applications

FASTQSim has been used to generate datasets for MIT Lincoln Laboratory’s Bioinformatics Challenge Days as well as the Defense Threat Reduction Agency’s challenge for the evaluation of available metagenomic algorithms [18]. In orchestrating both events, FASTQSim contributed to the generation of several truth datasets using the four next generation sequencing platforms (Illumina Hi-Seq, Ion Torrent PGM, PacBio, Roche 454). The tool was used to address two challenges. The first involved quality control of datasets generated across the different platforms. The second focused on the generation of custom datasets with target organisms and genes present at specific concentrations. The resulting datasets were used to evaluate performance of leading metagenomic analysis algorithms. One of the *in silico* datasets contained reads from seven source organisms, six bacteria and one virus. The dataset composition is described in Table 2).

**Table 2:** **Composition of Illumina** ***in silico*** **dataset generated with FASTQsim for the DTRA metagenomic algorithm challenge**

Organism taxonomy	Number of reads	Number of genes
Bacteria, Proteobacteria, Gammaproteobacteria, Thiotrichales, Francisellaceae, Francisella, tularensis.	206	163
[Genbank:NC_008245.1]		
Bacteria, Proteobacteria, Alphaproteobacteria, Rhizobiales, Methylobacteriaceae, Methylobacterium	148	110
radiotolerans JCM 2831. [Genbank:CP001001.1]		
Bacteria, Proteobacteria, Gammaproteobacteria, Pseudomonales, Pseudomonadaceae, Pseudomonas	201	101
aeruginosa pao1. [Genbank:NC_002516.2]		
Bacteria, Actinobacteria, Actinobacteridae, Actinomycetales, Corynebacterinae, Mycobacteriaceae,	200	111
Mycobacterium avium complex (mac). [Genbank:EU854994.1]		
Bacteria, Firmicutes, Bacilli, Lactobacillales, Streptococcaceae, Streptococcus pneumoniae ATCC 700669.	201	119
[Genbank:NC_011900.1]		
Bacteria, Proteobacteria, Gammaproteobacteria, Legionellales, Legionellaceae, Legionella pneumophila	50	37
Philadelphia 1. [Genbank:NC_002942.5]		
Human immunodeficiency virus I. [Genbank:NC_001802.1]	5	4

**Figure 7:**
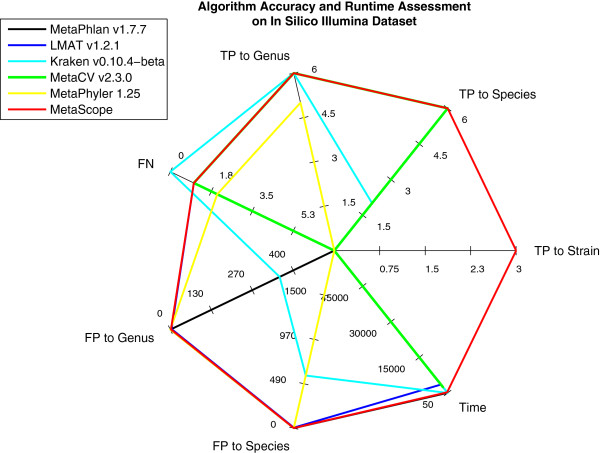
**Six leading metagenomic algorithms were evaluated on an** ***in silico*** **Illumina dataset generated with FASTQspike.** True positive and false positive algorithm calls were compared at the genus, species, and strain levels.

The dataset was used to evaluate the speed and accuracy of six leading metagenomic algorithms: MetaPhlan [19], Kraken [20], MetaPhyler [21], MetaCV [22], LMAT [23], and MetaScope [18]. Results are summarized in Figure 7. The *in silico* dataset with known truth enabled analysis of false positive and false negative error rates across the algorithms.

FASTQSim was also used to simulate datasets for the MITLL Bioinformatics Challenge Day, which required participants to identify and interpret the signatures of genetic engineering in a metagenomics dataset. A human whole blood background was sequenced with the IonTorrent platform^a^. Instructions for accessing the resulting FASTQ file are provided in Additional file 2. The BLAST algorithm was used to characterize the background dataset.

The target sequences for *in silico* inclusion underwent a series of modifications to model a scenario in which the E. coli K-12 MG1655 mutator strain was used as a host for the Enterobacteria phage lambda [Genbank:J02459.1]. The host E. coli strain had several genes deleted and several others turned off with multiplex automated genome engineering (MAGE) to accelerate the evolution of the phage [24]. The phage itself was subjected to codon substitution and rearranging to replace the codons *AGT*, *AGC*, *AGA*, *AGG*, *TAG*, *TTA*, *TTG* with other codons for the corresponding amino acids [25]. The modified phage was spliced into the E. coli genome, and FASTQSim was used to insert the engineered E.coli strain into the human blood background at a concentration of 17 ×. FASTQSim was used to spike the BioBrick cloning vector pSB3C5-I52001 [Genbank:EU496103.1] into the human blood background at a concentration of 12 × relative to the E. coli organism. The resulting dataset consisted of 46% reads generated with FASTQSim. Instructions for accessing the individual source files and the combined file generated for the Challenge Day by FASTQSim are provided in Additional file 2.

The Newbler assembler was used to measure the performance of the FASTQSim tool [26]. Reference assembly of the blended dataset was performed against the three sources of spiked reads. The results of the assembly are summarized in Table 3. The spiked organisms were present in the blended dataset in the expected quantities.

**Table 3:** **Newbler characterization for high coverage** ***in silico*** **IonTorrent dataset**

Reference accession	Num unique	Pct of all unique	Pct of	Pct coverage	Description
	matching reads	matches	all reads	of reference	
NC_000913.2	225797	98.04%	42.2%	97.50%	Escherichia coli str. K-12 substr. MG1655,
					complete genome
gi |215104|gb |J02459.1	2872	1.26%	0.50%	98.72	Enterobacteria phage lambda, complete
					genome, with codon substitution
EU496103.1	1543	0.70%	0.30%	100.0%	BioBrick cloning vector pSB3C5-I52001,
					complete sequence

## Conclusions

The emergence of next-generation sequencer technology has greatly increased the number and scope of genomic sequencing projects. Gigabases of data can be generated in a few hours, demanding rapid and accurate analysis algorithms and software. However, there are currently limited options to produce individual, simulated test cases for algorithm benchmarking. FASTQsim is a platform-independent tool for producing simulated read data sets, useful for designing sequencing projects and for testing and comparing bioinformatics or assembly software. Inputs to FASTQsim can be adapted to generate user defined sequence sets that can serve as verified example data.

## Availability and dependencies

FASTQSim is available to download from SourceForge:

-  Project name: FASTQSim
-  Project home page: https://sourceforge.net/projects/fastqsim
-  Operating system: Linux
-  Programming language: Shell scripts, Python
-  Other requirements:

– Python v.2.7 http://www.python.org/download/releases/2.7.5/

– matplotlib v.1.1.0+ http://matplotlib.org/downloads.html

– numpy v.1.7.1+ http://www.scipy.org/install.html

– BLAST 2.2.26+ ftp://ftp.ncbi.nlm.nih.gov/blast/executables/blast+/LATEST/

– Java Blast Parser (included) v.1.0

– samtools v.0.1.19+

-  License: GNU GPL v.3

A gzipped tar archive of the FASTQsim source code, README, and license is provided in Additional file 3. A shell executable script is provided in Additional file 4 to demonstrate the functionality of the FASTQsim software. Links for obtaining the necessary data files to execute the example are provided in Additional file 2.

## Endnote

^a^ Datasets for the study were supplied by Dr. C Nicole Rozenzweig at the Edgewood Chemical/Biological Center.

## Funding

This work is sponsored by the Defense Threat Reduction Agency under Air Force Contract # FA8721-05-C-0002. Opinions, interpretations, recommendations and conclusions are those of the authors and are not necessarily endorsed by the United States Government.

## Electronic supplementary material

Additional file 1: **JavaBlastParser java executable.** This is a java.jar file for the JavaBlastParser dependency. The file can be executed by running the “javac” command. (ZIP 21 KB)

Additional file 2: **List of data files for FASTQsim example.** This is a.txt text file that lists links to all data files necessary for executing the example FASTQsim shell script. (TXT 57 bytes)

Additional file 3: **FASTQsim source code.** This is a tar.gz archive of the FASTQsim source code, README, and license. (ZIP 11 MB)

Additional file 4: **Shell script for FASTQsim example.** This is a.sh shell executable script demonstrating the functionality of the FASTQsim software. (ZIP 801 bytes)
